# Inhibition of Programmed Death Receptor-1/Programmed Death Ligand-1 Interactions by Ginsenoside Metabolites

**DOI:** 10.3390/molecules25092068

**Published:** 2020-04-29

**Authors:** Nam-Hui Yim, Young Soo Kim, Hwan-Suck Chung

**Affiliations:** Korean Medicine (KM) Application Center, Korea Institute of Oriental Medicine (KIOM), 70 Cheomdan-ro, Dong-gu, Daegu 41062, Korea

**Keywords:** immune checkpoint blockade, PD-1/PD-L1, competition assay, ginsenosides, Rg3, Compound K

## Abstract

Evidence suggests that programmed death receptor-1/programmed death ligand-1 (PD-1/PD-L1) targeted inhibitors act as an immune checkpoint blockade, indicating that these compounds may be useful in cancer immunotherapy by inhibiting the immune response between T-cells and tumors. Previous studies have shown that ginsenosides can regulate the expression of PD-1 and PD-L1 in target diseases; however, it remains unknown whether ginsenosides act as a blockade of PD-1/PD-L1 interactions. In this study, we used competitive ELISA to investigate 12 ginsenosides for their ability to block PD-1/PD-L1 interactions. In addition, we performed a protein–ligand docking simulation and examined the hydrophobic interactions and hydrogen bonds formed at the interfaces between the ginsenosides and PD-L1/PD-1. Eight out of the 12 ginsenosides studied showed inhibition of PD-1/PD-L1 interactions at 35% at the maximum concentration (1 μM). Among them, Rg3 and Compound K (C-K) demonstrated the highest inhibitory effects. Rg3 and C-K were further identified for their interaction efficacy with PD-1/PD-L1, which supported our results demonstrating the blocking activity of these compounds against PD-1/PD-L1 binding interactions. Collectively, our findings suggest that some ginsenosides, including Rg3 and C-K, inhibit PD-1/PD-L1 binding interactions. Therefore, these compounds may prove useful as part of an overall immuno-oncological strategy.

## 1. Introduction

Studies of potential anticancer compounds that elicit an immune checkpoint blockade (ICB) have shown tangible results during a short period. Indeed, the recent research and development of immune checkpoint inhibitors, including monoclonal antibodies (mAbs) and small molecules for ICB-based immunotherapy, represents a major advance toward controlling a growing number of malignancies using immunosuppressive mechanisms for cancer treatment [1,2].

Programmed death receptor-1/programmed death ligand-1 (PD-1/PD-L1) are well-known targets for ICB. PD-1 plays an important role in the immune system as a regulator, particularly in the tumor microenvironment [3,4]. PD-1 is expressed in various immune-related cells, including T-cells, B-cells, and natural killer cells. PD-1 negatively regulates T-cell activity at the immune response stage by interacting with PD-L1 and PD-L2 through phosphatase activity. Through these interactions, PD-1 inhibits the kinase signaling of the associated ligand pathways, which serves to suppress T-cell activation. PD-L1 can be identified on activated T- and B-cells, dendritic cells (DCs), macrophages, and other tissue cells, whereas PD-L2 expression is limited to DCs, macrophages, and some stromal cells [1,4].

mAbs have been shown to block the effects of PD-1/PD-L1 interactions, resulting in a durable antitumor response and long-term remission in certain patients with a broad spectrum of cancers, including lymphoma, melanoma, lung cancer, bladder cancer, and renal cancer [3]. However, mAb therapy is high-priced and has numerous disadvantages, such as the immunogenicity, non-oral bioavailability rate, poor penetration of solid tumor tissues, poor pharmacokinetic control, and immune-related adverse events (irAEs) given their long half-life. In contrast, small molecules have shorter half-lives and can provide therapeutic alternatives to mAbs. In general, small molecules have increased oral bioavailability and bioefficiency, along with decreased immunogenicity [5].

Ginsenosides comprise a class of naturally-developed steroid glycosides and triterpene saponins derived from the plant genus *Panax*. To date, based on the identification of 70 triterpenoid saponins from ginseng, these compounds can generally be divided into protopanaxadiol (PPD) and protopanaxatriol (PPT) types, termed PPD-type ginsenosides (PDS) and PPT-type ginsenosides (PTS), according to their phytochemical skeletons. Ginsenosides have been extensively investigated for their anticancer activities, including their ability to inhibit cancer cell proliferation, angiogenesis, invasion, and metastasis, as well as their ability to regulate tumor-related immune suppression as the major anticancer components of ginseng [6,7]. While the structure–activity relationships of ginsenosides for their anticancer effects have been investigated, these compounds have not been fully investigated for their ability to elicit ICB.

In this study, we evaluated the binding inhibition of eight kinds of PDS and four kinds of PTS against PD-1 and PD-L1 interactions.

## 2. Results and Discussion

**Figure 1:**
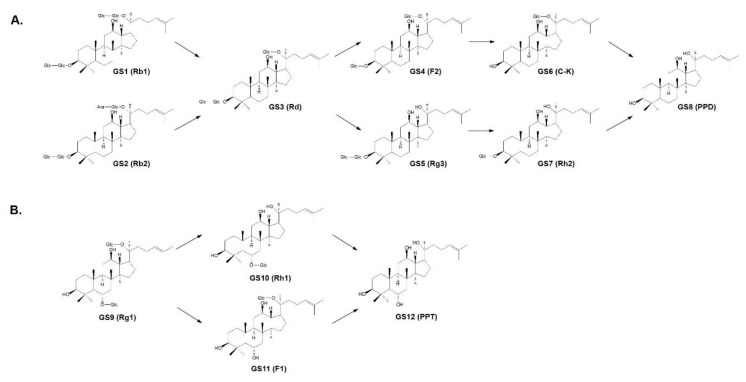
Metabolic processes of (**A**) protopanaxadiol-type ginsenosides (PDS) and (**B**) protopanaxatriol-type ginsenosides (PTS). GS1, Rb1; GS2, Rb2; GS3, Rd; GS4, F2; GS5, Rg3; GS6, Compound K (C-K); GS7, Rh2; GS8, protopanaxadiol (PPD); GS9, Rg1; GS10, Rh1; GS11, F1; GS12, protopanaxatriol (PPT).

**Figure 2:**
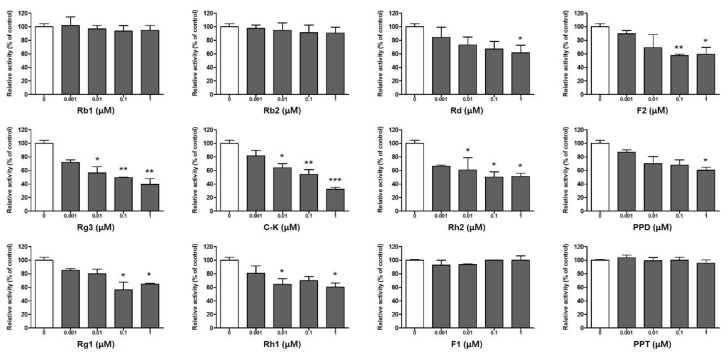
Inhibition of the programmed death receptor-1/programmed death ligand-1 (PD-1/PD-L1) binding interaction by ginsenosides using PD-1/PD-L1 competitive ELISA. Twelve ginsenosides were tested at 0.001, 0.01, 0.1, and 1 μM, and the inhibitory rate of ginsenosides against PD-1/PD-L1 was evaluated using a PD-1/PD-L1 Inhibitor Screening Assay Kit. Data are shown as the means ± SD of three independent experiments. * *p* < 0.05, ** *p* < 0.01, and *** *p* < 0.001, compared with blank (0) group.

Many studies have demonstrated that PD-1/PD-L1 targeted therapy provides a promising cancer immunotherapy approach through the inhibition of multiple stages of the immune response between T-cells and tumors. In particular, PD-L1 can be upregulated in accordance with infiltrating immune cells and the changing microenvironment within tumors, suggesting that it may prove to be a biomarker of cancer immunotherapy [1,4]. In order to identify the inhibitors of PD-1/PD-L1 binding interactions, we applied an assay system that detects the blocking ratio in competition with PD-1. In this assay, 12 ginsenosides were evaluated using biotin-labeled PD-1 to determine whether these compounds may be candidates for inhibiting PD-1/PD-L1 binding interactions. In general, PDS and PTS have a low rate of absorption in the intestines as a result of their hydrophilicity. Thus, upon oral intake, PDS and PTS are transformed into the small metabolites PPD and PPT, respectively, through deglycosylation and acid hydrolysis catalyzed by intestinal bacteria [8]. Among the eight kinds of PDS (Figure 1A), ginsenosides Rd (GS**3**), F2 (GS**4**), Rg3 (GS**5**), and C-K (GS**6**) are mainly detected in human plasma [9,10]. In addition, C-K has been found in the healthy volunteers’ blood treated with Rb1 (GS**1**), confirming the metabolic processing of ginsenosides [11]. Four kinds of PTS are present in the transformation pathway from Rg1 (GS**9**) to PPT (GS**12**) (Figure 1B). Ginsenoside Rg1 and its metabolites are also biotransformed via deglycosylation in the intestine and detected in plasma; however, the indicated amounts are very low because of the poor oral bioavailability of Rg1, which results from the limited membrane permeability and active biliary excretion of Rg1 [12]. Based on the structural features of the ginsenoside metabolites, we analyzed the individual efficacy of 12 ginsenosides for inhibiting PD-1/PD-L1 binding interactions using a competition assay. In addition, to ensure the reliability of this method, the inhibitory efficacy of the PD-1 neutralizing antibody directly binding with PD-L1 was confirmed as a positive control (Supplementary Materials Data Figure S1). As shown in Figure 2, almost all ginsenosides, including Rd, F2, Rg3, C-K, Rh2, PPD, Rg1, and Rh1 (from GS3 to GS10), showed an ability to block PD-1/PD-L1 interactions at 35% at the maximum concentration (1 μM), while four ginsenosides, Rb1, Rb2, F1, and PPT, showed no inhibition. Interestingly, the competitive inhibition of PD-1/PD-L1 interactions was stronger for the metabolic products compared to the parent ginsenosides Rb1 and Rb2. In particular, Rg3 and C-K showed significantly high inhibitory rates of PD-1/PD-L1 interaction in a dose-dependent manner at 60.4% and 67.7%, respectively, at 1 μM. These results indicated that ginsenoside metabolites may be potent ICB candidates against PD-1/PD-L1 interactions.

**Figure 3:**
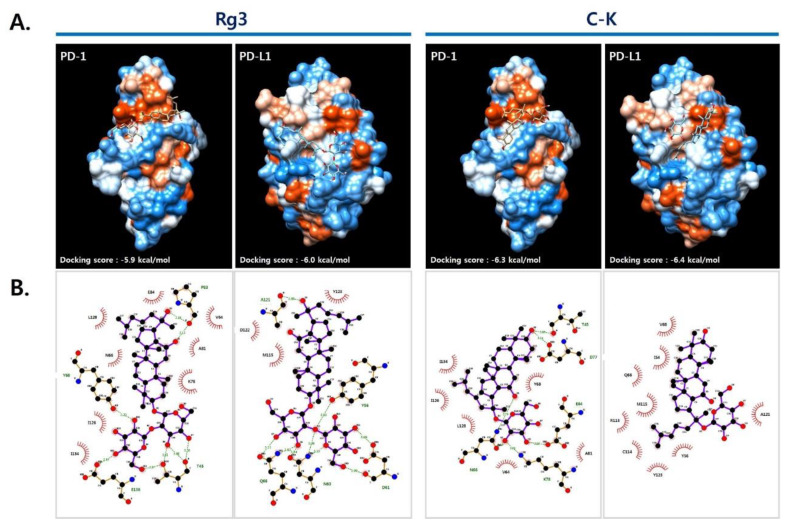
Protein–ligand docking simulation between PD-L1/PD-1 and Rg3/C-K. (**A**) Rg3 and C-K were docked into PD-1 and PD-L1 derived from the PD-1/PD-L1 complex (PDB code: 4ZQK) using AutoDock Vina. (**B**) The amino acids involved in hydrogen bonds and hydrophobic interactions between PD-L1/PD-1 and ginsenosides were presented via analysis using LigPlot+ v.1.4.5 (EMBL-EBI, Cambridge, UK). The amino acid residues involved in the interactions were indicated with black (hydrophobic interactions) and green (H-bonds).

Based on the above results, we performed molecular docking in order to further identify the interactions between PD-1/PD-L1 and the ginsenosides Rg3 and C-K on the basis of the crystal structures of the PD-1/PD-L1 complex (PDB code: 4ZQK) [13] and the PD-1/PD-L1 small molecule complex [14]. As shown in Figure 3A, in silico modeling of Rg3 and C-K into the PD-1/PD-L1 binding pocket resulted in higher docking scores for C-K, for both PD-1 and PD-L1 (−6.3 and −6.4 kcal/mol, respectively), compared with Rg3 (−5.9 and −6.0 kcal/mol, respectively). Using pharmacophore analysis, we further investigated the associated hydrophobic interactions and hydrogen bonds formed at the interfaces between PD-1/PD-L1 and the ginsenosides with LigPlot+ software (Figure 3B). Rg3 formed four hydrogen bonds (i.e., T45, Y68, P83, and E136) and eight hydrophobic interactions (i.e., V64, N66, K78, A81, E84, I126, L128, and I134) with PD-1 and five hydrogen bonds (i.e., Y56, D61, N63, Q66, and A121) and three hydrophobic interactions (i.e., M115, D122, and Y123) with PD-L1. Several amino acids in PD-1 (or PD-L1), associated with the interaction with Rg3, have been reported to play important roles in PD-1/PD-L1 interaction (PD-1, V64, N64, Y68, K78, I126, L128, and E136; PD-L1, Y56, Q66, M115, A121, D122, and Y123) [13]. C-K also formed five hydrogen bonds (i.e., N66, T76, D77, K78, and E84) and six hydrophobic interactions (i.e., V64, Y68, A81, I126, L128, and I134) with PD-1 and nine hydrophobic interactions (i.e., I54, Y56, Q66, V68, R113, C114, M115, A121, and Y123) with PD-L1. In particular, a number of amino acids in PD-1 (V64, I126, L128, and I134) and PD-L1 (I54, Y56, R113, M115, A121, and Y123) were found to interact with C-K and were involved in the hydrophobic interactions required to form the PD-1/PD-L1 complex. These results confirmed that C-K is a promising inhibitor of PD-1/PD-L1 interactions.

In recent clinical studies and clinical trials, it has been revealed that multiple additional co-inhibitory pathways by single agents or combination treatment lead to significant anticancer effects by affecting various immune response stages [15]. Despite the significant success in the treatment of various malignancies, antibody drugs have a high rate of irAEs, with multiple immune-related adverse reactions observed in immune-cancer therapy. Therefore, immunotherapeutic approaches have recently focused on the development of small molecules that can overcome the negative effects of antibody drugs [16,17]. In one previous study, Rg3 reduced PD-L1 expression by cisplatin resistance and resumed the cytotoxicity of cancer cells by activating T-cells in non-small-cell lung cancer (NSCLC) cells [18]. Furthermore, it has been confirmed that Rg3 can attenuate chemotherapeutic resistance, improving the efficacy of chemotherapy and prolonging the survival of patients with NSCLC [19]. These significant studies indicated that ginsenosides may regulate the expression of PD-1 and PD-L1 in target diseases; however, these studies did not demonstrate the function of ginsenosides as inhibitors of PD-1/PD-L1 binding interactions.

Although we applied the competition assay for screening PD-1/PD-L1 targeted inhibitors, on the basis of these results, we plan to apply other screening approaches in future research, including the antagonist-induced dissociation assay (AIDA) NMR, homogenous time-resolved fluorescence (HTRF), and surface plasmon resonance (SPR). In addition, further study of the active ginsenosides in cellular and animal-based studies of the PD-1/PD-L1 axis is warranted.

## 3. Materials and Methods

### 3.1. Ginsenosides

Twelve ginsenosides (i.e., Rb1, Rb2, Rd, F2, Rg3, Compound K (C-K), Rh2, PPD, Rg1, Rh1, F1, and PPT) were purchased from Faces Biochemical Co., Ltd. (Wuhan, China).

### 3.2. Competitive ELISA

In order to test whether a compound blocked the interaction between PD-1 and PD-L1, a PD-1/PD-L1 Inhibitor Screening Assay Kit was used, following the manufacturer’s instructions. Briefly, 1 μg/mL recombinant human PD-L1 (BPS Bioscience, #71104) was coated in a 96-well plate overnight. After that, the plate was washed 3 times with phosphate-buffered saline (PBS) containing 0.1% Tween (PBS-T) and blocked for 1 h at room temperature with PBS-T containing 2% (*w*/*v*) bovine serum albumen, followed by another wash. After washing, the samples were added or an anti-PD-1 neutralizing antibody was added as a positive control and incubated for 1 h. Biotinylated hPD-1 (BPS Bioscience, #71109) was added for 2 h at room temperature. After washing with PBS-T, diluted streptavidin–horseradish peroxidase (HRP) was added to each well while shaking at a low speed for 1 h. After washing with PBS-T three times, HRP substrates A and B were added. Relative chemiluminescence was measured using a SpectraMax L Luminometer (Molecular Devices, San Jose, CA, USA).

### 3.3. Docking Simulation and Interaction Analysis

Ginsenosides were docked onto the binding pockets of PD-1 and PD-L1 obtained from the PD-1/PD-L1 complex (PDB code: 4ZQK) and information in a previous report using AutoDock Vina integrated with UCSF Chimera Alpha v.1.13 (RBVI, San Francisco, CA, USA) [13,20]. The binding affinity between PD-L1/PD-1 and each ginsenoside was expressed as the lowest energy score in the binding simulation. The hydrophobic and hydrogen bonding interactions between PD-1/PD-L1 and the ginsenosides were analyzed using LigPlot+ v.1.4.5 (EMBL-EBI, Cambridge, UK) [21]. The amino acid residues involved in the interactions were indicated with black (hydrophobic interactions) and green (H-bonds).

### 3.4. Statistical Analysis

All data are presented as the mean ± standard deviation (SD). One-way analysis of variance (ANOVA) was used for multiple comparisons (GraphPad Prism v.5.03 for Windows; GraphPad Software Inc., San Diego, CA, USA).

## 4. Conclusions

In this study, we evaluated the inhibitory activity of ginsenoside metabolites for blocking PD-1 and PD-L1 interaction. Among 12 ginsenosides, eight kinds of PDS strongly inhibited the PD-1/PD-L1 binding interaction compared to four kinds of PTS. Especially Rg3 and C-K remarkably blocked the competitive PD-1/PD-L1 interaction, which confirmed the hydrophobic interaction with the individual amino acids in PD-1 and PD-L1, respectively, through pharmacophore analysis. Therefore, these findings suggest that ginsenosides, including Rg3 and C-K, inhibit PD-1/PD-L1 binding interactions, which may prove useful as part of an overall chemo-immune treatment strategy.

## Supplementary Materials

The following are available online. Figure S1: Inhibition of the PD-1/PD-L1 binding interaction by PD-1 neutralizing antibody (NTAb) using PD-1/PD-L1 competitive ELISA. * *p* < 0.05, *** *p* < 0.001 compared with blank (0) group.

## Abbreviations

Immune checkpoint blockade, ICB; programmed death receptor-1/programmed death ligand-1, PD-1/PD-L1; monoclonal antibodies, mAbs; immune-related adverse events, irAEs; protopanaxadiol, PPD; protopanaxatriol, PPT; PPD-type ginsenosides, PDS; PPT-type ginsenosides, PTS; antagonist-induced dissociation assay, AIDA; homogenous time-resolved fluorescence, HTRF; surface plasmon resonance, SPR; dendritic cells, DCs; Compound K, C-K; phosphate-buffered saline, PBS; horseradish peroxidase, HRP; standard deviation, SD; non-small-cell lung cancer, NSCLC.

## References

[1] Postow M.A., Callahan M.K., Wolchok J.D. (2015). Immune Checkpoint Blockade in Cancer Therapy. J. Clin. Oncol..

[2] Wang Y., Wu L., Tian C., Zhang Y. (2018). PD-1-PD-L1 immune-checkpoint blockade in malignant lymphomas. Ann. Hematol..

[3] Guan J., Lim K.S., Mekhail T., Chang C.C. (2017). Programmed Death Ligand-1 (PD-L1) Expression in the Programmed Death Receptor-1 (PD-1)/PD-L1 Blockade: A Key Player Against Various Cancers. Arch. Pathol. Lab. Med..

[4] Wilson R.A.M., Evans T.R.J., Fraser A.R., Nibbs R.J.B. (2018). Immune checkpoint inhibitors: New strategies to checkmate cancer. Clin. Exp. Immunol..

[5] Barakat K. (2017). Rational Design of Small Molecule Immune Checkpoints’ Inhibitors: The PD-1 Challenge. J. Pharm. Pharm. Sci..

[6] Bai L., Gao J., Wei F., Zhao J., Wang D., Wei J. (2018). Therapeutic Potential of Ginsenosides as an Adjuvant Treatment for Diabetes. Front. Pharm..

[7] Liu K.K., Wang Q.T., Yang S.M., Chen J.Y., Wu H.X., Wei W. (2014). Ginsenoside compound K suppresses the abnormal activation of T lymphocytes in mice with collagen-induced arthritis. Acta Pharmacol. Sin..

[8] Chen X.J., Zhang X.J., Shui Y.M., Wan J.B., Gao J.L. (2016). Anticancer Activities of Protopanaxadiol- and Protopanaxatriol-Type Ginsenosides and Their Metabolites. Evid.-Based Complement. Altern. Med..

[9] Kim J.K., Kim J.Y., Jang S.E., Choi M.S., Jang H.M., Yoo H.H., Kim D.H. (2018). Fermented Red Ginseng Alleviates Cyclophosphamide-Induced Immunosuppression and 2,4,6-Trinitrobenzenesulfonic Acid-Induced Colitis in Mice by Regulating Macrophage Activation and T Cell Differentiation. Am. J. Chin. Med..

[10] Kim D.H. (2018). Gut microbiota-mediated pharmacokinetics of ginseng saponins. J. Ginseng Res..

[11] Kim J.K., Choi M.S., Jeung W., Ra J., Yoo H.H., Kim D.H. (2019). Effects of gut microbiota on the pharmacokinetics of protopanaxadiol ginsenosides Rd, Rg3, F2, and compound K in healthy volunteers treated orally with red ginseng. J. Ginseng Res..

[12] He C., Feng R., Sun Y., Chu S., Chen J., Ma C., Fu J., Zhao Z., Huang M., Shou J. (2016). Simultaneous quantification of ginsenoside Rg1 and its metabolites by HPLC-MS/MS: Rg1 excretion in rat bile, urine and feces. Acta Pharm. Sin. B.

[13] Zak K.M., Kitel R., Przetocka S., Golik P., Guzik K., Musielak B., Domling A., Dubin G., Holak T.A. (2015). Structure of the Complex of Human Programmed Death 1, PD-1, and Its Ligand PD-L1. Structure.

[14] Magiera-Mularz K., Skalniak L., Zak K.M., Musielak B., Rudzinska-Szostak E., Berlicki L., Kocik J., Grudnik P., Sala D., Zarganes-Tzitzikas T. (2017). Bioactive Macrocyclic Inhibitors of the PD-1/PD-L1 Immune Checkpoint. Angew. Chem. Int. Ed..

[15] Adams J.L., Smothers J., Srinivasan R., Hoos A. (2015). Big opportunities for small molecules in immuno-oncology. Nat. Rev. Drug Discov..

[16] Sasikumar P.G., Ramachandra M. (2018). Small-Molecule Immune Checkpoint Inhibitors Targeting PD-1/PD-L1 and Other Emerging Checkpoint Pathways. Biodrugs.

[17] Han Y., Gao Y.N., He T., Wang D.D., Guo N., Zhang X.T., Chen S.Z., Wang H. (2018). PD-1/PD-L1 inhibitor screening of caffeoylquinic acid compounds using surface plasmon resonance spectroscopy. Anal. Biochem..

[18] Jiang Z., Yang Y., Yang Y., Zhang Y., Yue Z., Pan Z., Ren X. (2017). Ginsenoside Rg3 attenuates cisplatin resistance in lung cancer by downregulating PD-L1 and resuming immune. Biomed. Pharm..

[19] Sun Y., Lin H., Zhu Y., Feng J., Chen Z., Li G., Zhang X., Zhang Z., Tang J., Shi M. (2006). A randomized, prospective, multi-centre clinical trial of NP regimen (vinorelbine + cisplatin) plus Gensing Rg3 in the treatment of advanced nonsmall cell lung cancer patients. Zhongguo Fei Ai Za Zhi.

[20] Pettersen E.F., Goddard T.D., Huang C.C., Couch G.S., Greenblatt D.M., Meng E.C., Ferrin T.E. (2004). UCSF chimera - A visualization system for exploratory research and analysis. J. Comput. Chem..

[21] Laskowski R.A., Swindells M.B. (2011). LigPlot+: Multiple Ligand-Protein Interaction Diagrams for Drug Discovery. J. Chem. Inf. Model..

