# Supplementary material for: Inhibition of Programmed Death Receptor-1/Programmed Death Ligand-1 Interactions by Ginsenoside Metabolites
Source: Molecules. 2020 Apr 29;25(9):2068. doi: 10.3390/molecules25092068 (PMC7249111; doi:10.3390/molecules25092068)
Supplement: Supplementary file 1 [file molecules-25-02068-s001.pdf]

molecules

MDPI

Supplementary Materials:

Inhibition of Programmed Death Receptor-1/Programmed Death Ligand-1 Interactions by Ginsenoside Metabolites

Nam-Hui Yim, Young Soo Kim and Hwan-Suck Chung *

Korean Medicine (KM) Application Center, Korea Institute of Oriental Medicine (KIOM), 70 Cheomdan-ro, Dong-gu, Daegu 41062, Republic of Korea; nhyim23@kiom.re.kr (N.-H.Y.); yskim527@kiom.re.kr (Y.S.K.)

* Correspondence: hschung@kiom.re.kr; Tel.: +82-53-940-3875; Fax: +82-53-940-3899

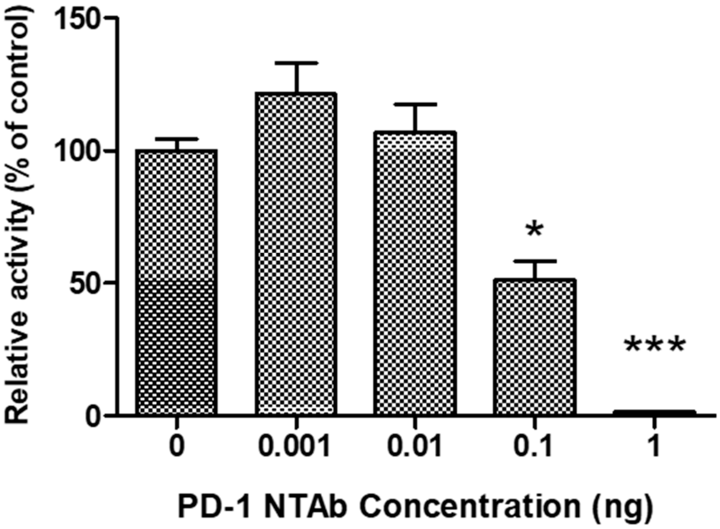

Figure S1: Inhibition of the PD-1/PD-L1 binding interaction by PD-1 neutralizing antibody (NTAb) using PD-1/PD-L1 competitive ELISA. * $p < 0.05$, *** $p < 0.001$ compared with blank (0) group.
